# Appendicitis Caused by Primary Varicella Zoster Virus Infection in a Child with DiGeorge Syndrome

**DOI:** 10.1155/2017/6708046

**Published:** 2017-08-16

**Authors:** Lotte Møller Smedegaard, Claus Bohn Christiansen, Linea Cecilie Melchior, Anja Poulsen

**Affiliations:** ^1^Department of Clinical Microbiology, Copenhagen University Hospital, Rigshospitalet, Blegdamsvej 9, 2100 Copenhagen, Denmark; ^2^Department of Pathology, Copenhagen University Hospital, Rigshospitalet, Blegdamsvej 9, 2100 Copenhagen, Denmark; ^3^Department of Paediatrics and Adolescent Medicine, Copenhagen University Hospital, Rigshospitalet, Blegdamsvej 9, 2100 Copenhagen, Denmark

## Abstract

**Introduction:**

Chickenpox is caused by varicella zoster virus (VZV). Although predominantly a mild disease, it can cause considerable morbidity and in rare occasions even mortality in healthy children as well as increased morbidity and mortality in immunocompromised patients. The aetiology of appendicitis is largely unknown but is thought to be multifactorial. Appendicitis is a suspected, but not well documented, complication from varicella zoster virus infection.

**Case Presentation:**

A five-year-old girl diagnosed with DiGeorge syndrome and a prolonged primary VZV infection was admitted due to abdominal pain, increasing diarrhoea, vomiting, and poor general condition. She developed perforated appendicitis and an intraperitoneal abscess. VZV DNA was detected by PCR in two samples from the appendix and pus from the abdomen, respectively. The child was treated with acyclovir and antibiotics and the abscess was drained twice. She was discharged two weeks after referral with no sequela.

**Conclusion:**

Abdominal pain in children with viral infections can be a challenge, and appendicitis has to be considered as a complication to acute viral diseases, especially if the child is immunocompromised.

## 1. Introduction

Chickenpox is caused by varicella zoster virus (VZV). Although predominantly a mild disease, it can cause considerable morbidity and on rare occasions even mortality in healthy children as well as increased morbidity and mortality in immunocompromised patients. The most common complications are bacterial skin infections, mild hepatitis, and mild thrombocytopenia [1].

The aetiology of appendicitis is largely unknown but is thought to be multifactorial. The most frequent cause is generally accepted to be luminal obstruction, of which the most common causes are probably fecaliths and hyperplasia of the lymphoid follicles. Bacterial, parasitic, and viral infections can all lead to lymphoid hyperplasia [1]. Autopsy studies have shown intranuclear inclusions in both lymphoid tissue and the gastrointestinal tract in patients with disseminated VZV infection [2]. It is therefore likely that VZV infection can cause appendicitis, but this is not well documented.

In this report we describe a child who developed a perforated appendix and an intraperitoneal abscess during a prolonged primary VZV infection.

## 2. Case Presentation

A five-year-old girl diagnosed with DiGeorge syndrome at the age of 5 months with thymic hypoplasia and decreased T-cell counts was transferred to our department from a secondary paediatric department. She had been admitted the day before with worsening diarrhoea, vomiting, and malaise. She had contracted varicella two weeks prior to her admission and still had fresh vesicles covering most of her body on arrival, indicating a prolonged primary VZV infection. Two months prior to infection she was considered to have a close to normal T-cell function and a normal B-cell function. On admission, a chest X-ray had shown pneumonic infiltration, and she was being treated with intravenous antibiotics and acyclovir. C-reactive protein (CRP) steadily began to decline from 250 to 210 mg/l and white cell count (WCC) from 17.7 to 15.6 × 10^9^/l (normal age-adjusted range 4.5–12.5 × 10^9^/l) of which segmented neutrophils declined from 14.2 to 11.7 × 10^9^/l (normal age-adjusted range 1.8–8.9 × 10^9^/l).

An acute abdomen was suspected, and a CT-scan revealed possible free air and fecaliths, indicating a likely perforated appendix. It was at this point that she was transferred to our department.

On the basis of the CT-scan and the clinical findings of guarding and severe abdominal pain in the right lower quadrant, an open laparotomy was performed and a gangrenous perforated appendix was removed. Histopathological examination showed an appendix coated with fibrin and segmented neutrophils with increased wall thickness, transmural acute necrotising inflammation, and distal perforation. Postoperatively the intravenous antibiotic treatment with meropenem, ciprofloxacin, metronidazole, and acyclovir was continued. The WCC normalised within the first postoperative day (POD) and CRP declined to 127 × 10^9^/l on the second POD.

**Figure 1:**
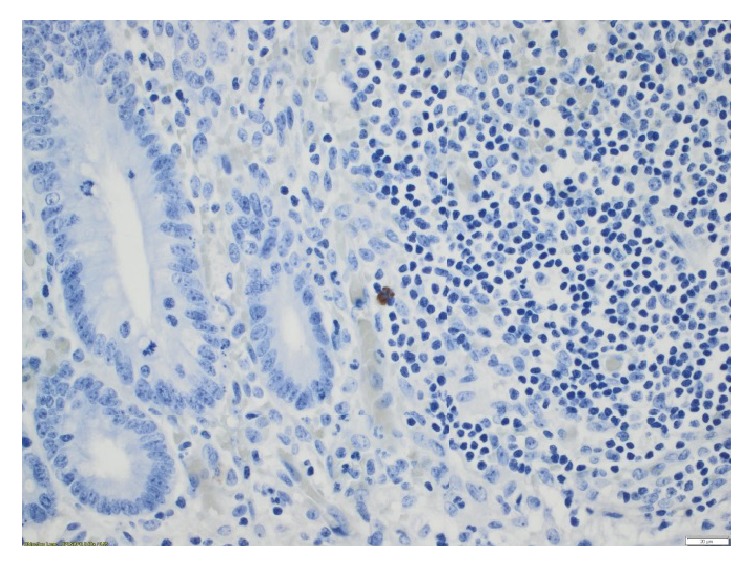
Positive result of immunostaining for varicella zoster virus (VZV) in mucosa [×40].

**Figure 2:**
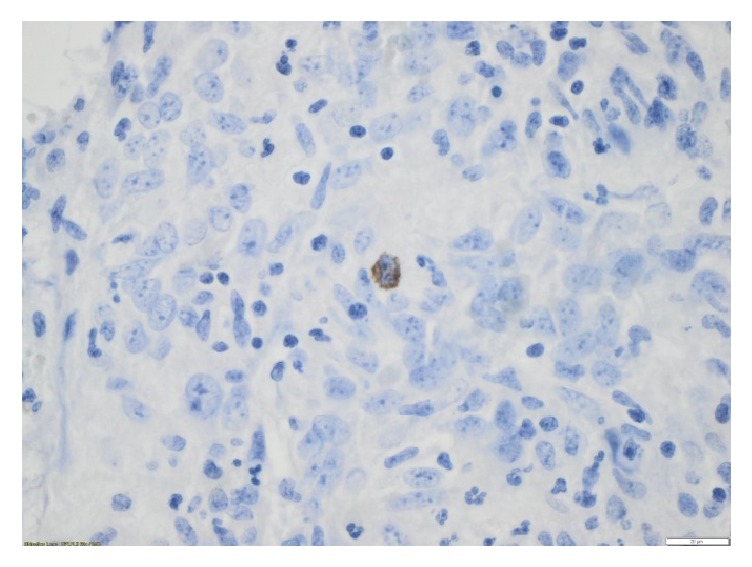
Submucosa [×60]. Red-brown staining was regarded as a positive result.

On the fifth POD the WCC started to rise again, and the patient developed a fever of over 39°C. On clinical examination her abdomen was tense in the right lower quadrant. An ultrasound showed an intraperitoneal localised fluid collection, which was emptied with ultrasound guided technique. Microscopy of the pus showed a few gram positive rods, and* Clostridium cadaveris* was found in the culture. A polymerase chain reaction (PCR) assay detecting VZV DNA identified VZV in DNA material isolated from two tissue samples from the appendix (Figures 1 and 2) and in one sample of pus from the abdomen. PCR for VZV DNA in the patient's saliva was negative. On the tenth POD the WCC rose again, and an ultrasound showed a recurrence of the abscess. This was subsequently incised, and while no fluid was extracted, the patient improved quickly. On POD thirteen, CRP had declined to 13 × 10^9^/l and acyclovir was stopped. The child was discharged on POD fourteen, and antibiotic treatment was continued with oral ciprofloxacin and amoxicillin for seven days after discharge. The parents cancelled a planned follow-up two weeks after discharge because the child was doing so well.

## 3. Discussion

Acute appendicitis is a common cause for abdominal surgery in children, but the aetiology is still largely unknown. It is thought to be multifactorial, but different studies have indicated that bacterial or viral infections can be associated with the condition. In the most recent study by Richardsen et al. from 2016, they found* E*.* coli* as the far most predominant bacteria, but also streptococci,* Pseudomonas aeruginosa*, and* Yersinia* species. Adenovirus and rotavirus were the most common viral pathogens, although they were more rare causes of appendicitis [3]. We have only found two studies regarding the association between VZV infection and appendicitis. A historical study from England examined the association between appendicitis and childhood infections such as varicella, measles, mumps, rubella, scarlet fever, and whooping cough and only found significant association between appendicitis and mumps [4]. In a newer study Katzoli et al. performed a systematic investigation of the appendices, blood samples, and part of the omentum of 38 children with acute appendicitis. PCR assays were used to detect herpes virus DNA in the specimens. None of the samples were VZV DNA positive, but in eight cases cytomegalovirus DNA was detected in all three samples, and in three of the cases humane herpes virus 6 (HHV-6) DNA was detected in all three samples [5]. This strongly suggests that viral infections may be associated with appendicitis in children. We have found scarce reports on appendicitis as a complication of primary VZV infection, and we only found one other case where VZV DNA was verified by PCR in the appendix [6].

It is well known that the risk of disseminated VZV infection and complications is increased in immunocompromised children, but previous cases have shown that appendicitis is a possible complication even in otherwise healthy and immunocompetent children [7].

In our patient, the positive PCR for VZV DNA from the appendix (Figures 1 and 2) and the pus in the abdominal abscess show that the appendicitis was most probably caused by VZV. The child was considered to have a close to normal T-cell count shortly before contracting chickenpox and was therefore most probably immunocompetent at the time.

More studies will be required to fully establish the role of viral infections and VZV specifically in the aetiology of appendicitis.

Abdominal pain in children with viral infections can be a challenge and paediatricians should be aware of the possibility of appendicitis as a complication of acute viral disease in children.
